# Aqueous Humor Outflow Structure and Function Imaging At the Bench and Bedside: A Review

**DOI:** 10.4172/2155-9570.1000578

**Published:** 2016-07-24

**Authors:** Alex S. Huang, Chirayu Mohindroo, Robert N. Weinreb

**Affiliations:** 1 Doheny Eye Institute, Los Angeles, CA, USA; 2 Department of Ophthalmology, David Geffen School of Medicine at UCLA, Los Angeles, CA, USA; 3 Hamilton Glaucoma Center and Shiley Eye Institute, University of California, San Diego, CA, USA

**Keywords:** Aqueous humor, Aqueous angiography, OCT, Glaucoma, Minimally invasive glaucoma surgery, Trabecular meshwork, Intraocular pressure

## Abstract

Anterior segment glaucoma clinical care and research has recently gained new focus because of novel imaging modalities and the advent of angle-based surgical treatments. Traditional investigation drawn to the trabecular meshwork now emphasizes the entire conventional aqueous humor outflow (AHO) pathway from the anterior chamber to the episcleral vein. AHO investigation can be divided into structural and functional assessments using different methods. The historical basis for studying the anterior segment of the eye and AHO in glaucoma is discussed. Structural studies of AHO are reviewed and include traditional pathological approaches to modern tools such as multi-model two-photon microscopy and optical coherence tomography. Functional assessment focuses on visualizing AHO itself through a variety of non-real-time and real-time techniques such as aqueous angiography. Implications of distal outflow resistance and segmental AHO are discussed with an emphasis on melding bench-side research to viable clinical applications. Through the development of an improved structure: function relationship for AHO in the anterior segment of the normal and diseased eye, a better understanding of the eye with improved therapeutics may be developed.

## Introduction and History

Glaucoma can result in irreversible vision loss. At this time, the only proven treatment is lowering intraocular pressure (IOP) [1,2]. IOP is elevated in glaucoma due to increased resistance to fluid outflow from the eye [3,4]. Methods for IOP lowering include topical medicines, lasers, and invasive surgeries. Today, a new category of glaucoma surgery, Minimally Invasive Glaucoma Surgeries (MIGS) [5,6], is of great interest. MIGS offer a small wound, safe, and fast surgical approach to enhance aqueous humor outflow (AHO) drainage. With some of the early MIGS procedures, there has been inconsistent success [7–10]. Therefore, the importance of furthering our understanding of how aqueous humor exits from the eye is of increasing interest. Using new and emerging technologies, AHO structure and function is being re-examined with a goal of improving current and enhancing future therapeutics.

To understand the full AHO outflow pathways, one has to understand how it was discovered. Historically, one of the earliest clues for AHO came from Lauber’s dogs. Taking blood from ciliary veins, it was noted that the ciliary vein blood hematocrit was less than the peripheral vein hematocrit [11]. This resulted in the hypothesis that the eye was diluting the blood by exuding some fluid into the systemic circulation leading to the discovery of the trabecular/conventional outflow pathway. While today we know that two AHO pathways exist (trabecular/conventional and uveoscleral/unconventional) [12–15], the purpose of this review is to focus on the conventional AHO route and the role of MIGS.

**Figure 1:**
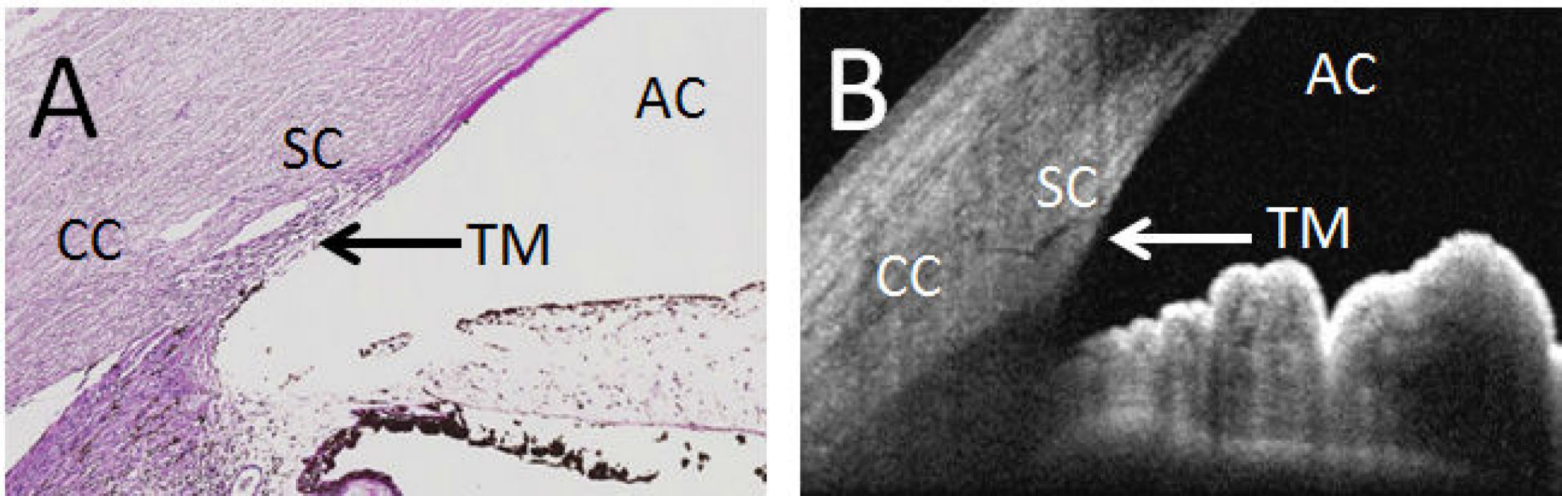
The Conventional Aqueous Humor Outflow Pathway. **(A)** Angle and aqueous humor outflow pathways can be visualized by histology with hematoxylin and eosin staining or by **(B)** Optical Coherence Tomography (taken using Heidelberg Spectralis HRA+OCT Anterior Segment Module from a living individual). AC: Anterior Chamber; TM: Trabecular Meshwork; SC: Schlemm’s Canal; CC: Collector Channels.

To figure out the physical structures that carried aqueous, outflow pathway mapping was done via casting experiments. Polymerizing agents such as neoprene latex or vulcanizing silicone were injected retrogradely into distal outflow structures identified either by direct visualization or anterior filling with trypan blue [16,17]. Enzymatic removal of surrounding ocular tissue resulted in beautiful branching three-dimensional casts denoting the potential AHO anatomy. These casts, in addition to histology (Figure 1), suggested that aqueous humor flowed from the anterior chamber, passed the trabecular meshwork (TM) into Schlemm’s Canal (SC) through collector channels, into an intrascleral venous plexus, and eventually to aqueous and episcleral veins [3,4,18]. While fundamentally critical and illuminating regarding basic anatomy, casting studies were limited based on their static (non-real-time) assessments of AHO and concerns that results were non-physiologic because of high and “steady” pressure required to push polymerizing agents through the eye [17].

To complement structural studies, seminal aqueous humor dynamic physiological experiments were conducted by Morton Grant and others [4]. Using a basic aqueous humor perfusion rig, outflow facility was measured in both normal and glaucomatous enucleated human eyes before and after TM removal [4]. We have re-organized Morton Grant’s results into one table based on outflow resistance to better summarize the results. First, the TM was the primary resistor for AHO (~70–75% incorporating data from Morton Grant and others [19,20]) (Table 1; blue arrow). Second, glaucoma eyes showed more AHO resistance compared to normal eyes (Table 1; green arrow). These two observations provided the basis for conventional AHO research and clinical thought in normal and glaucomatous eyes for the next half-century. Changes to extracellular matrix [21], cytoskeletal changes [22], secreted pro-fibrotic factors [23], theories of funneling [24], and TM biomechanics [25] have all contributed to a model explaining impaired outflow at the level of the TM.

However, careful examination of the AHO resistance measurements resulted in two more observations. First, even after TM removal, some level of resistance was still recorded implying potential sources of resistance distal to the TM (Table 1; ”Resistance after Trabeculotomy” values). Second, glaucoma eyes showed greater post-TM resistance values (2.53 mm Hg × min/microliters) compared to normal eyes (1.26 mm Hg × min/microliters) (Table 1; red arrow) implying that the post-TM AHO regions were also effected in glaucoma. In fact, the increase in TM (Table 1; green arrow; 2.7 fold) and post-TM (Table 1; red arrow; 2 fold) resistance in glaucoma were nearly equivalent. This suggested that glaucoma was not only a TM outflow resistance problem but potentially a whole-eye disease where mechanisms to explain impaired outflow would have to explain both TM and post-TM changes.

Minimally invasive glaucoma surgeries (MIGS) and their results from the last decade have re-invigorated glaucoma anterior segment thought and research. Trabecular MIGS moved from basic goniotomies/trabeculotomies [26,27] to using electrocautery or bypass shunts to overcome TM-related resistance by connecting the anterior chamber directly to Schlemm’s canal [5,6]. While IOP lowering has occurred, inconsistent results have naturally led to further consideration of either post-TM resistance seen in the physiologic experiments above (Table 1; green and red arrows) or a re-evaluation of AHO patterns [7–10].

This review surveys AHO research with an emphasis on the entire conventional outflow pathway, trabecular and post-trabecular. Research will be roughly organized into balancing the study of AHO structure (anatomy) and function (physiology of fluid flow) (Table 2). Glaucoma clinicians are already accustomed to structural (optical coherence tomography) and functional (visual field) relationships in the posterior segment. Our goal is to delineate the relationship between structure and function for the anterior segment as well.

## Structural Assessment of Aqueous Humor Outflow

Today, understanding AHO structure is more sophisticated than traditional casting methods. Initially, pathological/histological and electron microscopic methods were used [18]. TM, SC, and distal outflow structures were observed. Sampling of these structures across the eye revealed an overall impression of non-360 degrees uniform luminal sizes and presence [28]. Electron microscopic results implied the presence of “plaque” material in glaucomatous eyes not found in normal eyes that may have been related to increased outflow resistance. Limitations of these approaches were associated with variability in tissue procurement, possible fixation artifacts, and sample preparations differences [24,25,29].

Recently, structural assessments, particularly of the TM, have occurred in a more native three-dimensional context with two-photon multimodal imaging. Two photon-microscopy (TPM) enabled high-resolution deep tissue optical sectioning in whole fixed or live tissue [30]. Different modalities, such as autofluoresence and second harmonic generation demonstrated native trabecular anatomy and allowed for segregation of different extracellular matrix components such as collagen and elastin [31–33]. This type of tissue-based trabecular analyses has led to exploration of live cell dynamics and study of drug effects for IOP modulation. TPM of AHO anatomy, however, is currently not applied in living human individuals.

To evaluate post-TM AHO structures, multiple approaches have been used. Three-dimensional micro-computed tomography (3D micro-CT) in enucleated eyes identified AHO pathways as lumens of low radiographic signal [34]. SC, collector channels, and large intrascleral vessels were successfully identified. Results 360 degrees around the limbus resembled AHO outflow casts with approximately 24–29 channels counted.

To study AHO structure in living subjects, anterior segment optical coherence tomography (OCT) has been indispensable. Like 3D micro-CT, AHO lumens appeared as regions of low reflectivity on OCT scans (Figure 1). Spectral domain OCT (SD-OCT) successfully identified the TM, SC, collectors channels and intrascleral venous plexus. Image processing allowed for creation of three-dimensional *in silico* casts of small segments of distal AHO pathways in normal individuals [35]. Pilocarpine in live patients (Skaat et al. IOVS 2014; 55: ARVO Abstract 5682) and mice has been reported to increase the size of these AHO lumens while IOP elevation has been shown to do the opposite [36,37]. Automated segmentation has been proposed (Murakami Y et al. IOVS 2014; 55: ARVO Abstract 927).

Phase-based OCT (ph-OCT) has shown pulse-dependent TM motion in enucleated non-human primate and live human eyes [38,39]. In live human imaging, there was significant correlation with the digital/cardiac pulse with TM motion. This correlation allowed assessment of the phase lag and time delay between TM motion and the cardiac pulse where the digital pulse would occur prior to the TM motion. A significant linear relationship was also present between the TM phase lag and the heart rate.

While illuminating, current imaging-based structural analyses of AHO pathways were limited by understanding the relevance of the anatomy observed. It was unclear if large lumens necessarily represent increased or decreased AHO. The trabecular meshwork itself, oft described as a hypo-reflective interface shadow could connect to SC and be confusingly included as a portion of the AHO pathways [40]. Structure-to-structure correlation of OCT to histology in the anterior segment has not yet reached the stage of what has been done in the posterior segment and retina [41–43]. Therefore, the biological relevance of the AHO pathway motion is unclear.

Also, most OCT studies have focused on sampling portions of the outflow anatomy [35]. While sampled images have demonstrated bead-like and segmental structures, full 360 degree outflow mapping in a live person is still needed.

## Functional Assessment of Aqueous Humor Outflow

Since the structure of a physiologic system often controls and predicts its function, without an exact working relationship between the two, the meaning of structural variations can on its own be unclear. Thus, functional assessment sometimes takes the primary role in patient care especially because it is usually the function of the organ that is what's clinically relevant. Glaucoma physicians are accustomed to this for posterior segment evaluation. Visual fields are a functional assessment of the structure (retinal ganglion cells as assessed by histology or OCT). Visual fields and defects therein are what a glaucoma patient actually experiences, as opposed to the absolute number of ganglion cells or thickness of the retinal nerve fiber layer on an OCT scan. Thus for glaucoma diagnosis and management, functional assessment of vision with visual fields historically preceded that of more structural evaluations that eventually became prominent today. Recent improvements in posterior segment structure and function relationship was required for this to happen [41–43].

For AHO, functional assessment is equal to assessing the flow itself. This can be determined experimentally with outflow facility measurements in enucleated eyes or calculated from clinically measured variables with help from the Goldmann equation [3]. Simultaneously, another functional assessment of AHO is to actually visualize where and how aqueous humor flows in the eye. This is important as, instead of considering the eye as one single unit of outflow, regional variations can be tested and visually appreciated. Imaging of AHO can be divided into non-real time and real time methods.

## Non Real-Time Techniques for Functional Assessment of Aqueous Humor Outflow

Non-real time methods to visualize AHO means adding a tracer to the eye, waiting a set period of time, and then capturing this information, typically in an enucleated eye after fixation for histological or whole mount study. Labeled tracers can include gold particles, fluorescent microspheres, or quantum dots (Qdots) among others.

Gold particles or cationic ferritin were initially used with electron microscopic analyses [44–46]. Segmental uptake of gold particles was seen at the cellular level for non-human primate inner-wall SC cells [44]. Biological relevance was demonstrated through a conversion of segmental into a more homogenous pattern using a serine-threonine kinase inhibitor (H-7 (1-(5-isoquinoline sulfonyl)-2-methyl piperazine)) known to block actomyosin-driven contractility [44].

For a more global AHO assessment, larger and fluorescently tagged tracers were delivered into either live or enucleated eyes in several species. Fluorescent microspheres ranged in size from 0.2 to 20 microns [22,44,47–51]. Alternatively, 0.01 micron quantum dots were used [52]. Imaging was done with laboratory fluorescent or confocal microscopes. Segmental patterns were seen, and combining tracer studies with histology, estimates of TM utilization could be calculated from a percent effective filtration length [48]. Two color tracer studies allowed for demonstrating reduced AHO with increasing IOP [53]. Tracer collection in the TM localized near collector channels in enucleated human eyes [47].

To probe the underlying biology associated with segmental AHO, biochemical and genetic studies were performed. Regions of high and low tracer accumulation could be isolated in mouse and probed by immunofluorescence [52]. RT-PCR demonstrated increased versican (a large extracellular matrix proteoglycan) levels in low flow regions. Quantitative PCR arrays in enucleated human eyes demonstrated alterations in several extracellular matrix genes and their regulatory proteases [51]. Using mouse models, segmental localization of fluorescent tracers in the TM of wild-type mice was abolished in the matricellular secreted protein acidic and rich in cysteine (SPARC) mutant [22]. Distally, fluorescein microspheres could be seen past SC in the mouse as well [22].

Taken together, non-real time imaging techniques have emphasized the segmental nature of physiologic AHO and a potential relevance for treating ocular hypertension. Particularly exciting is the link of the segmental patterns to possible protein differences that might subserve the segmentalization. Note though that emphasis was on the TM here. Some of this was due to the use of larger particles. Larger particles allowed better maintained intraluminal presence without leakage so that tracers stayed within the AHO pathways during the processing time necessary for the non-real time assessment. However, larger particles may have also had a harder time passing through the TM easily. Thus, while clearly important for understanding AHO biology in model systems, non-real-time approaches using tagged beads or tracers may have a more limited clinical use in live human subjects and patient care.

## Real-time Techniques for Functional Assessment of Aqueous Humor Outflow

Real-Time AHO imaging refers to visualizing tracer movement without the requirement to stop and process the tissue. The potential advantage here is that the variable of time between tracer introduction and tissue processing for non-real-time assessment is avoided. For the most part, real-time techniques have focused on small and clinically available tracers (fluorescein, indocyanine green (ICG), or trypan blue) using more clinical and surgical techniques. This is likely because clinical assessment of AHO in patient care would be better real-time since non-real-time methods rely so much upon pathological and histological methods.

Channelography and canalography are essentially the same technique that relies on unroofing Schlemm’s canal (SC) with introduction of the tracer (fluorescein, ICG, or trypan blue) [54–59]. The introduction can occur through local injection analogous to a viscocanulostomy surgery or through intermittent deposition from a flexible microcatheter as it is pulled through the eye during canaloplasty surgery. Such techniques have demonstrated images of AHO distinguishing ocular surface vessels associated with AHO from those that are not. While highlighting the anatomy, such approaches focus more on the potential space where aqueous humor can move. By introducing the tracer in SC, TM contributions are not considered. Also, no matter how quickly a surgeon pulls a flexible microcatheter out of the eyes 360 degrees around the limbus, it is impossible to deliver tracer simultaneously circumferentially to fairly assess segmental differences. Lastly whether the tracer is delivered at uniformly physiological pressures is unclear if the tracer is introduced while a microcatheter is actively being exited from the eye [60].

Searching for clear episcleral fluid waves represented another clever approach to identifying AHO pathways by looking for negative signal in the form of disappearance of blood in surface epislceral veins [61,62]. Conducted with the irrigation function on commercially available phacoemulsification units during cataract surgery, by delivering a clear perfusate into the anterior chamber, local AHO pathways were determined by observing the flushing out of episcleral veins. The advantage is that this technique would be very familiar and comfortable to ophthalmic surgeons. Disadvantages are the need to look for loss of signal as opposed to positive signal and the high/supra-physiologic pressures used. Clinically, when studied retrospectively, a statistically significant correlation was seen between patients with extrinsic episcleral fluid waves and better surgical success with trabecular bypass [62].

**Figure 2:**
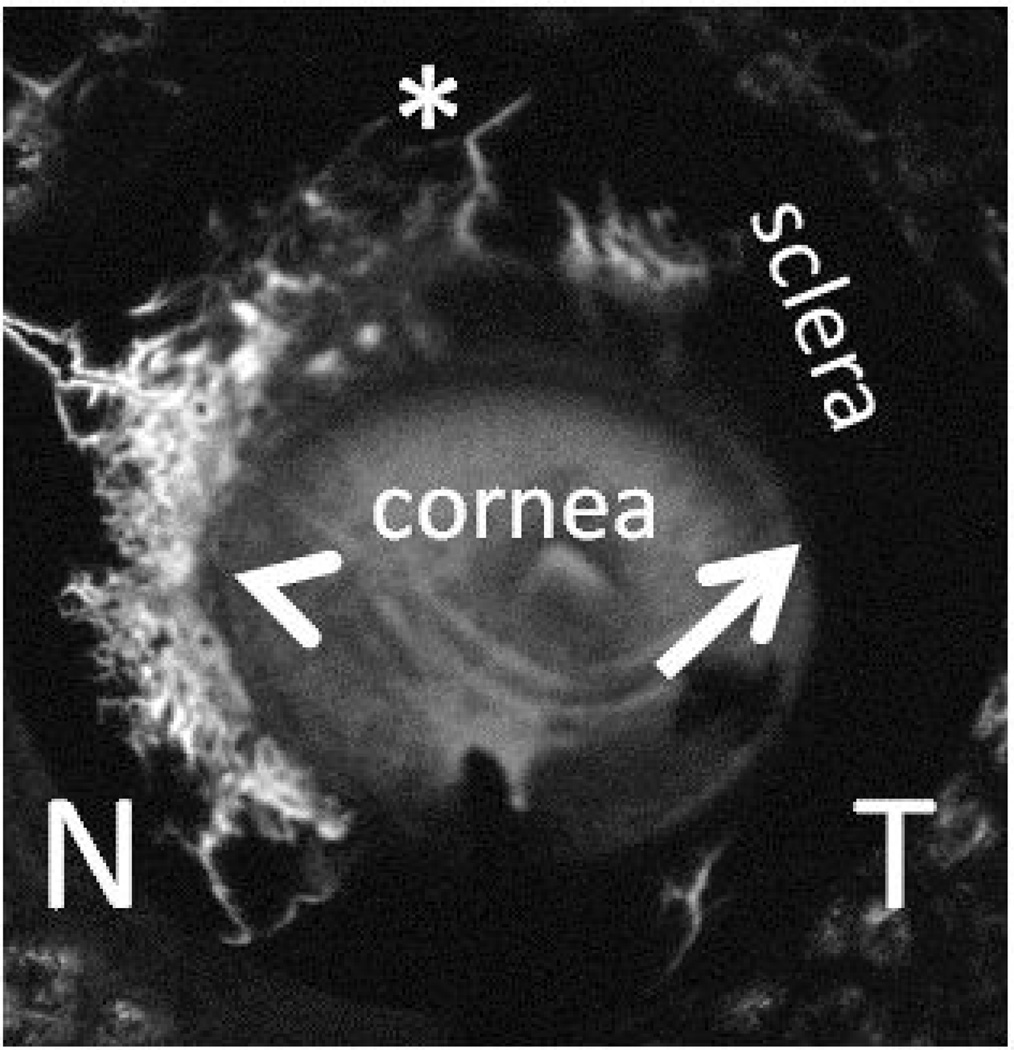
Aqueous angiography visualizes segmental aqueous humor outflow. Aqueous angiography was performed in the post-mortem left eye of a 79 year old female who died of cardiopulmonary arrest and not known to have ophthalmic disease. The eye was orientated face-on. 2.5% fluorescein diluted in balanced salt solution was delivered into the anterior chamber by an interior anterior chamber maintainer and outflow patterns imaged by the Heidelberg Spectralis HRA+OCT. Arrowhead points out peri-limbal regions of angiographic signal or flow, arrow points out regions without, and asterisk points out distal signal. N: Nasal and T: Temporal.

More recently, combining different aspects of the above approaches, aqueous angiography was a method that delivered fluorescent tracers into the anterior chamber with external imaging using an experimental microscopic setup [63] or a clinically FDA-approved angiographic device [60] (Heidelberg Spectralis HRA+OCT) (Figure 2). Using enucleated human and pig eyes, physiologic pressures were used, and introduction of the tracer into the anterior chamber allowed for simultaneous tracer delivery circumferential around the eye so that segmental changes could be fairly assessed. More importantly, delivery into the anterior chamber allowed outflow and angiographic results to be influenced by the TM.

Aqueous angiography was so termed to distinguish it from canalograms because the approach and information provided was different. This is analogous to the difference between classical congenital glaucoma surgeries: goniotomies and trabeculotomies [26,27]. For aqueous angiography and goniotomies, ab-interno approaches were taken. Instead, canalography and trabeculotomies used ab-externo approaches. While goniotomies and trabeculotomies achieved the same result of TM destruction, aqueous angiography further differed from canalography in that aqueous angiography provided real-time AHO imaging that included influence from the TM.

This difference in information also parallels the techniques of percutaneous transhepatic cholangiography (PTC) and endoscopic retrograde cholangiopancreatography (ERCP) where, as the names imply, both can visualize the biliary system but ERCP provides easier images of the exocrine pancreatic ducts to detect pancreatic pathologies [64,65]. Additionally, like canalograms and trabeculotomies, PTC could be considered ab-externo, as the tracer is introduced external through the surface skin inserting a needle into the gall bladder. ERCP, like aqueous angiography and goniotomies, takes an internal approach through the gastrointestinal lumen.

Using enucleated pig and human eyes, aqueous angiography demonstrated segmental AHO patterns that mimicked non-real-time methods. Angiographic signal was validated as representing true AHO using anterior segment OCT and fluorescent dextrans studies [60]. Angiographically positive but not negative regions demonstrated intrascleral lumens compatible with AHO on anterior segment OCT [60]. With fluorescent dextrans, angiographically positive but not negative regions demonstrated trapping of the tracer in AHO pathways in the TM and angle [60]. Quantitative methods have been developed to analyze these types of images [63].

## Choice of Tracer for Functional Assessment of Aqueous Humor Outflow

The meaning of any tracer study in any system is only as good as how close the tracer models the native substance of interest. The use of multiple tracers above with similar results of segmental AHO patterns in multiple species has been very reassuring. For AHO, aqueous humor is actually made up of many components [66]. Predominately, aqueous humor is composed of water (molecular weight=18 g/mol). Small components such as ions are present. Other, less abundant components include small molecules (eg vitamin C…), to macromolecules (protein/DNA/RNA), to larger structures and cellular components. In a way, testing AHO with multiple tracers of different characteristics is the best way to model AHO. Larger tracers such microspheres [47–52] may reflect the larger component of aqueous. Given ICG’s propensity for protein binding, ICG aqueous angiography (Saraswathy S, et al. IOVS 2015; 56: ARVO Abstract 247) may mimic flow of proteins. Smaller tracers such as fluorescein better models small molecules and water [60]. Putting these together a more comprehensive and realistic picture of aqueous outflow can be presented.

## Aqueous Humor Outflow Imaging and the Clinic

Clinically, aqueous humor outflow and its segmental nature may play a role in normal versus glaucomatous physiology and response to glaucoma therapy (medical or surgical). From a pharmacological standpoint, creating more uniform AHO may be beneficial [22,44,67].

From a surgical standpoint, AHO imaging may help guide and customize MIGS in the individual patient for optimized IOP lowering. However, even with segmental aqueous angiographic information, a common clinical question is whether trabecular MIGS should be targeted to regions of greater flow to take advantage of known AHO pathways. The downside is that if flow is good, potential improvement there may be limited. Alternatively, should surgery be performed where flow is bad with the hopes of recruiting additional and increased AHO? This would be analogous to surgically improved AHO uniformity. The potential disadvantage is that maybe flow in initially poor regions may have been low because the anatomy didn’t support AHO in the first place. Now, tools for structural and functional assessment are available to ask these questions. Early experiments suggested that regions of initially poor flow could be recruited with TM bypass using a trabecular bypass stent (Huang AS, et al, 2016; AGS: paper presentation PA23).

In patient care, combining many of the above techniques will be important. Ideally, clinical angiographic AHO assessment is real-time, includes contributions from the entire AHO pathways, allows for circumferential testing to fairly assess segmentalization and be conducted at physiologic pressures. For surgeries, real-world considerations must be had. While aqueous humor perfusion rigs have become ever sophisticated, patient safety, surgeon comfort, avoidance of unnecessary additional wounds, and time under anesthesia are factors. For example, most surgeons would not be willing to place a sharp needle in the sulcus space of a phakic patient to deliver the tracer there just because it is in a more physiologic starting point compared to the anterior chamber. Like the episcleral fluid wave approach, something familiar and comfortable to the ophthalmic surgeon will be important [61,62].

In conclusion, for glaucoma, creating posterior segment structure: function relationships have been underway and are already quite developed [41–43]. In the anterior segment, a separate relationship is lacking and needs to be had. Modern tools, including anterior segment OCT and aqueous angiography, will likely play a role in creating that anterior segment structure: function understanding. Clinical relevance may come in the form of developing new pharmacological tools using structural or functional endpoints or improving surgical results from custom-targeted surgical approaches.

## Figures and Tables

**Table 1:** Summary of Morton Grant’s Results. Outflow facility was taken from Grant [4]. Normal values (n=15) were obtained from Table 5 [4]. Open angle glaucoma values (n=6) were taken from Table 8 [4]. Outflow facility values were averaged and inverted to represent the data as resistance. This table organized measured resistance in normal and glaucoma eyes before and after trabeculotomy.

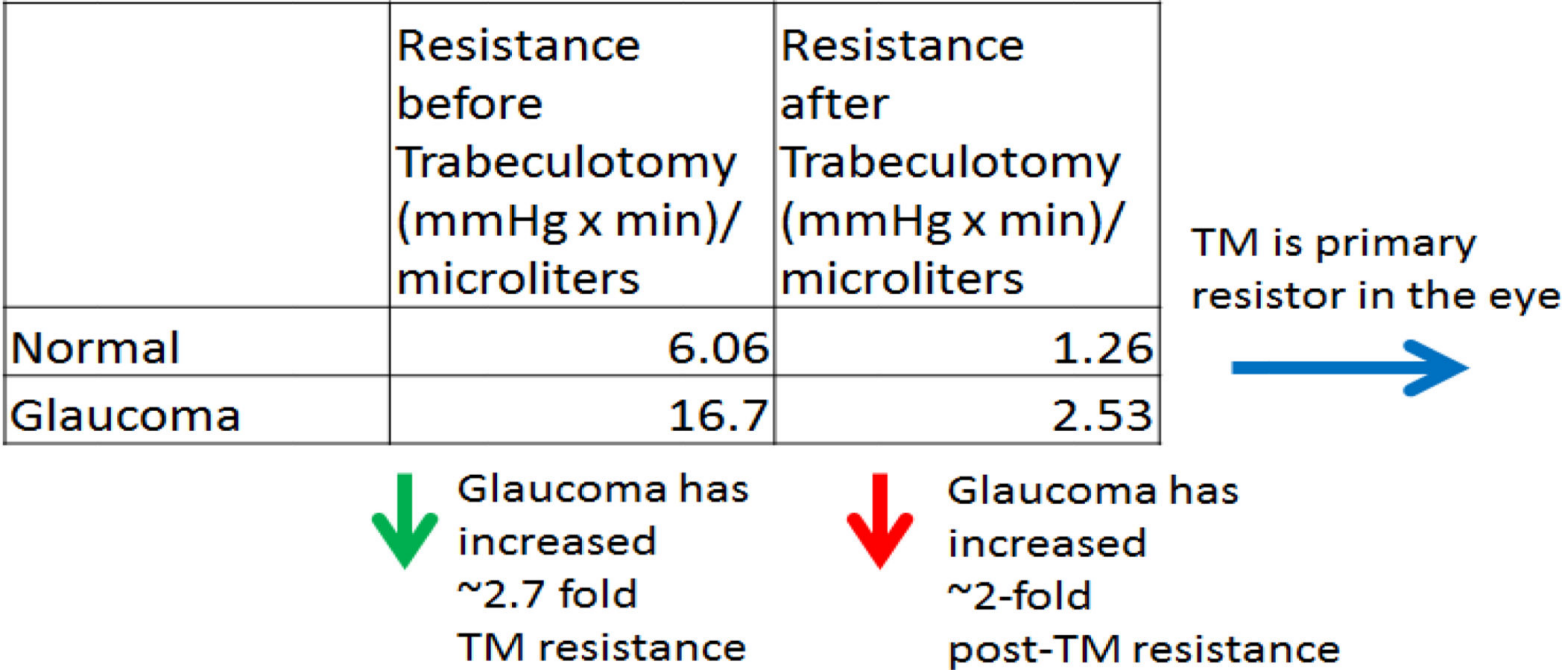

Blue Arrow: a drop in resistance in both normal and glaucoma eyes after trabeculotomy demonstrated that the primary resistor to outflow in the eye was the trabecular meshwork (TM). Green Arrow: comparing normal to glaucoma eyes, there was increased resistance to outflow in glaucoma eyes implying a resistance problem for the elevated intraocular pressure seen in glaucoma. Red Arrow: increased resistance to outflow was also seen in glaucoma eye after trabeculotomy (~2-fold) at a similar size to that determined to be from the TM (green arrow; ~2.7-fold) suggesting that underlying pathology in glaucoma affected the whole eye, TM and post-TM.

**Table 2:** Selected articles regarding structural and functional assessment of aqueous humor outflow focused on advanced ophthalmic imaging and tracer delivery studies.

Structural Assessment of Aqueous Humor Outflow		
Manuscript	Method	Key Findings
#^34^ Hann et al., 2011	3D micro-CT scanner	360 degrees AHO pathway mapping in an enucleated human eye.
#^35^ Kagemann et al., 2012	Anterior segment spectral-domain OCT	Sampled AHO pathway mapping in living human eyes.
#^39^ Li et al., 2014	Anterior segment phase-contrast OCT	AHO pathway pulsatile motion imaging in living human eyes.
**Non Real-time Functional Assessment of Aqueous Humor Outflow**		
#^47^ Chang et al., 2014	Fluorescent microspheres	Segmental TM tracer distribution in enucleated human eyes.
#48 Battista et al., 2008	Fluorescent microspheres	IOP-related decrease in effective filtration and increase in collector channel collapse in enucleated bovine eyes.
#^51^ Vranka et al., 2015 #^52^ Keller et al., 2011	Fluorescent microspheres	Changes in TM extracellular matrix proteins across areas of high or low segmental flow regions in enucleated human eyes.
**Real-time Functional Assessment of Aqueous Humor Outflow**		
#^56^ Grieshaber et al., 2010	Canalography	Episcleral vein visualization after direct tracer injection into Schlemm's canal during human surgery.
#^61^ Fellman et al., 2014	Episcleral Venous Fluid Wave	Outflow visualization after introduction of clear perfusate into anterior chamber during human surgery.
#^60^ Saraswathy et al., 2015	Aqueous Angiography	Real-time outflow visualization with intracameral tracer delivery into the enucleated porcine and human eyes.
